# Analysis of Endoplasmic Reticulum Stress-Associated Proteins As Prognostic Markers In Breast Cancer

**DOI:** 10.2174/0113892029374158251030064845

**Published:** 2026-01-06

**Authors:** Smriti Shreya, Shweta Pandey, Debasish Kumar Ghosh, Puneet, Shyam Babu Prasad, Christophe F. Grosset, Buddhi Prakash Jain

**Affiliations:** 1 Gene Expression and Signaling Lab, Department of Zoology, Mahatma Gandhi Central University, Motihari, Bihar, India;; 2 Department of Biotechnology, Govt Vishwanath Yadav Tamaskar Post-Graduate Autonomous College, Durg, Chhattisgarh, India;; 3 Kasturba Medical College, Manipal Academy of Higher Education, Manipal, Karnataka 576104, India;; 4 Department of General Surgery, Institute of Medical Sciences, Banaras Hindu University, Varanasi, Uttar Pradesh, India;; 5 Department of Zoology, Mahatma Gandhi Central University, Motihari, Bihar, India;; 6 MIRCADE Team, U1312, Bordeaux Institute in Oncology, BRIC, Université de Bordeaux, 146 Rue Léo Saignat, F-33000 Bordeaux, France

**Keywords:** Breast cancer, unfolded protein response, endoplasmic reticulum stress, prognostic marker, human protein atlas

## Abstract

**Introduction:**

Breast cancer is a complex, heterogeneous disease that poses a significant global health risk. Both internal and external cellular stresses contribute to breast cancer progression. Endoplasmic reticulum (ER) stress is one such cellular stress response that activates intricate intracellular signaling pathways collectively known as the unfolded protein response (UPR). Maintaining protein homeostasis and regulating these pathways is essential in breast cancer progression.

**Methods:**

Using STRING and Harmonizome Reactome pathway datasets, we identified a list of UPR-associated genes. The Human Protein Atlas and UALCAN databases were used to analyze these genes as potential prognostic markers in breast cancer.

**Results:**

Three prognostic markers were identified in patients with breast cancer: FK506 binding protein 14 (FKBP14), S-phase kinase-associated protein 1 (SKP1), and Baculoviral IAP repeat containing 3 (BIRC3).

**Discussion:**

Expression levels of FKBP14, SKP1, and BIRC3 were compared to TCGA normal and GTEx data using the GEPIA2 database. Our analysis indicates that higher SKP1 expression is associated with poor overall survival and prognosis, whereas higher BIRC3 expression correlates with better prognosis and overall survival. BIRC3 protein levels are elevated in tumor tissue and increase as the tumor progresses through various stages. Additionally, the expression of these markers varies according to sex, age, ethnicity, breast cancer subtype, nodal metastasis, and menopause status.

**Conclusion:**

Overall, our study identifies that the genes involved in ER stress that are associated with breast cancer can serve as prognostic markers.

## 1 INTRODUCTION

Breast cancer is one of the major diseases affecting women worldwide and is the most common type of malignancy globally. According to the latest GLOBOCAN report from the International Agency for Research on Cancer (IARC), as presented on the Global Cancer Observatory, breast cancer is the second leading cause of cancer after lung cancer [1]. Women are at a higher risk than men. Breast cancer is a multifactorial disease, with risk factors including age, obesity, diet, family history, and previous benign breast conditions. It arises from a complex interplay between genetic and environmental factors [2]. Preclinical and clinical studies indicate that these risk factors are associated with adipose tissue inflammation, angiogenesis, immune dysregulation, and extracellular matrix stiffness, all of which contribute to increased incidence and severity of breast cancer [3, 4].

A key factor in cancer development is the interaction of tumor cells with their microenvironment, which consists of immune cells, extracellular matrix, mechanical cues, and signaling molecules [5]. This interplay shapes the unique tumor microenvironment. During breast cancer progression, tumor cells experience various intracellular and extracellular stresses within this microenvironment [6]. Stress response pathways can enhance malignant properties, modify the tumor microenvironment, and suppress anti-tumor immune responses. Among these pathways, endoplasmic reticulum (ER) stress plays a critical role in cancer development [7, 8].

The ER is essential for proper protein folding, maintaining protein homeostasis, and performing post-translational modifications of membrane-bound and secreted proteins. In the tumor microenvironment, the protein-folding capacity of the ER is disrupted in both cancer and infiltrating immune cells, leading to an accumulation of unfolded and misfolded proteins [9, 10]. This phenomenon, known as ER stress, activates a complex signaling cascade called the unfolded protein response (UPR), which is regulated by three major sensors: protein kinase R-like ER kinase (PERK), inositol-requiring enzyme 1 (IRE1), and activating transcription factor 6 (ATF6) [10].

Under mild stress, UPR acts cytoprotectively, whereas under severe stress, it can trigger apoptosis. Therefore, the duration and intensity of stress determine the outcome of ER stress [11]. Elevated UPR levels have been observed in several cancers, including breast cancer [12, 13]. Breast cancer progression involves multiple stages, beginning with ductal hyperproliferation, followed by carcinoma *in situ*, invasive carcinoma, and ultimately metastatic disease. The increasing incidence and mortality of breast cancer highlight the need for advanced predictive biomarkers. Exploring the molecular mechanisms underlying breast cancer progression is crucial for identifying novel biomarkers that can improve diagnostics, staging, therapeutic assessment, and clinical management of patients.

FK506 binding protein 14 (FKBP14) is a multifunctional and widely conserved protein [14]. Members of the FKBP family bind to FK506 due to the presence of a highly conserved FK12-like domain, which defines their name and function. Each member also exhibits peptidyl-prolyl cis-trans isomerase activity, interconverting peptide bonds involving proline between cis and trans configurations [15, 16]. FKBP14 is involved in several cellular functions, including protein folding, stability, kinase activity, and T-cell activation [17, 18]. Mutations in this gene cause Ehlers-Danlos Syndrome (EDS) [19].

This protein is elevated in several malignancies and promotes tumor metastasis in ovarian, cervical, and osteosarcoma cancers. FKBP14 is overexpressed in cancers such as gastric cancer and is involved in cell proliferation and migration [20]. It also functions as a component of the IL8/JAK/STAT3 pathway, with JAK/STAT signaling playing a critical role in breast cancer migration [21]. FKBP14 catalyzes the proper folding of collagen type III and interacts with collagen types III, VI, and X [22]. Collagen has been implicated in tumor progression by regulating cytokines and associated signaling pathways [23]. FKBP14 also regulates presenilin proteins [24], which are transmembrane components of the γ-secretase complex and catalyze the cleavage of substrates, including the Notch receptor [25]. Notch signaling reprograms the tumor microenvironment and metabolism of cancer cells, including breast cancer [26].

S-phase kinase-associated protein 1 (SKP1) is a core component of the SCF (SKP1-Cullin 1-F-box protein) complex, an E3 ubiquitin ligase essential for ubiquitination of multiple protein targets [27]. SKP1 plays a critical role in regulating DNA damage response, cell cycle progression, and apoptosis. ER stress leads to decreased SKP1 expression at both mRNA and protein levels, resulting in accumulation of p27, which is not degraded proteasomally, thereby inducing G1 arrest [28]. SKP1 interacts with Bzip60 in UPR signaling and shows decreased expression in breast cancer, suggesting tumor suppressor-like functions [29, 30]. It also modulates BRCA1 protein levels and mediates the degradation of phosphorylated p27 [31], targeting proteins for proteasomal degradation.

Baculoviral IAP repeat-containing 3 (BIRC3) is a highly versatile member of the IAP family that regulates apoptosis, modulates inflammatory signaling, and influences immune responses. It also contributes to cell invasion and metastasis [32]. BIRC3 inhibits apoptosis by binding to tumor necrosis factor receptor-associated factors, TRAF1 and TRAF2. In contrast, prolonged activation of IRE1 promotes apoptosis through recruitment of TRAF2 [33]. ER stress can trigger the IRE1-TRAF2-JNK signaling pathway *via* IRE1-mediated TRAF2 phosphorylation [34]. BIRC3 has been shown to inhibit caspase activity, thereby acting as a pro-oncogenic, anti-apoptotic, and chemoresistance-promoting protein.

Several studies indicate that the unfolded protein response (UPR) is upregulated in various cancers. Using the Human Protein Atlas database, we performed a systematic analysis of UPR-associated proteins as prognostic markers in breast cancer. Among 165 ER stress-related proteins, we identified three, FKBP14, SKP1, and BIRC3, whose expression levels significantly correlated with prognosis in breast cancer patients.

## 2 METHODS

### 2.1 Selection of UPR Genes

A set of 165 proteins associated with the unfolded protein response (UPR) was curated using the Harmonizome and STRING databases [35, 36]. The Human Protein Atlas, a publicly available resource, was used to evaluate the prognostic significance of these proteins in patients with breast cancer. This analysis allowed the identification of potential prognostic markers among the 165 UPR-associated proteins. All data utilized in this study were derived from The Cancer Genome Atlas (TCGA) and GTEx, accessed on October 3, 2023, following the database guidelines [37]. Patient survival analyses were conducted for all 165 UPR-associated markers [38].

### 2.2 Expression analysis of selected prognostic markers using GEPIA and UALCAN

#### 2.2.1 GEPIA

The GEPIA (Gene Expression Profiling Interactive Analysis) dataset is a web-based tool that enables users to explore and compare RNA sequencing data from The Cancer Genome Atlas (TCGA) and the Genotype-Tissue Expression (GTEx) project. This platform allows analysis of RNA expression in breast cancer tissues compared to normal tissues [39]. In many cancer studies, normal tissues are often obtained from areas adjacent to tumors. Using GEPIA, we analyzed the expression levels of prognostic markers across breast cancer subtypes, performed differential gene expression analysis at different pathological stages, assessed correlations between gene expression and cancer prognosis, and conducted overall survival analyses. Tissue-specific expression of ER stress prognostic markers in breast cancer was visualized using dot plot analysis through the Expression DIY tool in GEPIA2.

#### 2.2.2 UALCAN

UALCAN platform provides a user-friendly interface for interpreting cancer-related data and helps to assess gene expression analysis, patient survival, and relevant molecular features [40]. UALCAN is also used to compare the expression levels of different genes across different subgroups. Analysis of UPR-associated genes in breast cancer about sex, age, race, metastasis status, and methylation is assessed using UALCAN [41, 42].

Requirements for boxplots are as follows:

Cancer stages – According to the American Joint Committee on Cancer (AJCC), samples are divided into stages I, II, III, and IV.

Patient age: Samples were grouped based on the age of patients, like 21 to 40, 41 to 60, 61 to 80, and 81 to 100 years.

Patient race: Samples were grouped into African-American, Caucasian, and Asian.

Menopause status: For breast cancer and endometrial Cancer, the menopause status of patients includes pre-menopause, peri-menopause, and post-menopause.

Patient gender: The patient's gender was classified as male or female.

Patient subclass: The subclass was divided into luminal, HER2-positive, and triple-negative breast cancer.

Nodal metastasis: Patients were grouped according to nodal metastasis as N0, N1, N2, and N3.

Supplementary Table **1** summarizes the number of participants/ samples for parameters in the bioinformatic analysis from different databases.

#### 2.2.3 Sample Collection

Fifty-six clinical specimens from breast cancer patients across stages I–IV were collected with patient consent following ethical approval from the Institute of Medical Sciences, BHU, India. All procedures were conducted in accordance with the guidelines of the Ethics Committee of IMS, BHU, for patients admitted between 2021 and 2023.

#### 2.2.4 Western Blotting

Cells were lysed in a lysis buffer containing 50 mM Tris-HCl (pH 7.4), 150 mM sodium chloride, 1 mM EDTA, 1% sodium deoxycholate, 0.1% SDS, 1 mM phenylmethylsulfonyl fluoride (freshly added), and protease inhibitors at 4°C for 30 minutes with intermittent vortexing. Protein concentrations were determined using a BCA assay, and 60 µg of protein from each sample was loaded onto a 12% SDS-PAGE gel for electrophoresis. Gels were run at 100 V until the dye front nearly reached the bottom. Proteins were then transferred onto a PVDF membrane using a dry-transfer system, with the membrane activated in 100% methanol prior to transfer.

Following transfer, membranes were blocked with 5% skim milk in TBS buffer (20 mM Tris-HCl, pH 7.6, 150 mM NaCl) for 1 hour at room temperature and subsequently washed three times for 5 minutes each with TBST (TBS + 0.1% Tween-20). Membranes were incubated overnight (12 hours) at 4°C on a rocker with primary antibodies against BIRC3 diluted in 5% skim milk in TBS. After incubation, membranes were washed three times with TBST for 5 minutes each, followed by incubation with secondary antibodies diluted in 5% skim milk in TBS for 2 hours at room temperature on a rocker. Membranes were then washed three times with TBST for 5 minutes each, followed by a final rinse with deionized water. Protein signals were detected using an HRP-based chemiluminescence system and visualized with a ChemiDoc imaging system.

## 3 RESULTS

### 3.1 Determining Prognostic Markers for Breast Cancer

Various proteins associated with ER stress and the unfolded protein response (UPR) have been implicated in cancer progression. An extensive analysis of UPR-associated proteins as prognostic markers in breast cancer was performed using the Human Protein Atlas. This study identified three genes, FKBP14, BIRC3, and SKP1, as prognostic markers out of 165 selected ER stress-related genes (Supplementary Table **2**). Among these, SKP1 and FKBP14 were associated with unfavorable outcomes, correlating with poor prognosis and increased risk of disease aggressiveness, whereas BIRC3 was linked to favorable outcomes and better prognosis (Table **1**). In the Human Protein Atlas, genes were classified as prognostic if the log-rank *P* value in maximally separated Kaplan-Meier analysis was less than 0.001, illustrating the association between high and low gene expression levels.

### 3.2 Expression Levels of Different Prognostic Markers

**Fig. (1):**
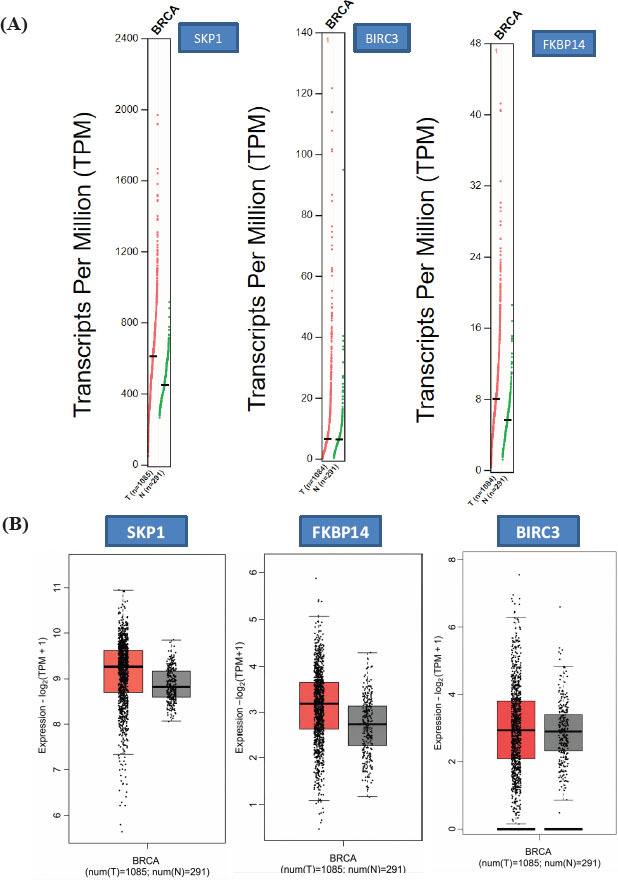
(**A**) SKP1, FKBP14, and BIRC3 expression level across BRCA TCGA cancer types (GEPIA2). Dot plots of SKP1, FKBP14, and BIRC3 differential expression levels in breast cancer patients (TCGA) compared to the normal (TCGA normal and GTEx). Each dot represents a distinct tumor (pink) or normal sample (green). The abscissa represents the sample from tumor tissue (T) or normal tissue (N), and “n” represents the sample size. The ordinate represents the amount of transcript expression in the sample, and the expression data were log2(𝑇𝑃M+1) transformed. Green coding indicates that the target gene has relatively lower expression in tumor tissue, and red indicates higher expression. (**B**) Differential expression of SKP1, FKBP14, and BIRC3 gene in BRCA and normal tissues in box plot using GEPIA2 database. The orange Box represents the tumor samples, while the grey box represents the normal tissues. GEPIA2; gene expression profiling interactive analysis 2, TPM; transcripts per million.

We compared the relative expression of the selected prognostic genes, FKBP14, BIRC3, and SKP1, by analyzing RNA sequencing data from breast cancer tissues and normal adjacent tissues. Using the GEPIA2 dataset, box plots were generated from GTEx data to assess gene expression levels in normal tissue. TPM values indicated that these three genes are differentially expressed between normal and tumor cells (Fig. **1a**). Box plot analysis, which visualizes median expression levels, interquartile ranges, and outliers, showed that SKP1 and FKBP14 are expressed at higher levels in tumor samples compared to normal adjacent tissues. In contrast, the median expression of BIRC3 was similar in both tumor and normal samples, suggesting that its expression change in tumors is not significant (Fig. **1b**). Heat map comparison of these genes revealed that SKP1 exhibits the highest expression among the three prognostic markers studied (Supplementary Fig. **S1**). Visualization through these tools enhances the understanding of gene expression patterns and highlights genes potentially involved in breast cancer progression.

### 3.3 Expression of SKP1, FKBP14, and BIRC3 in Different Stages of Breast Cancer

**Fig. (2):**
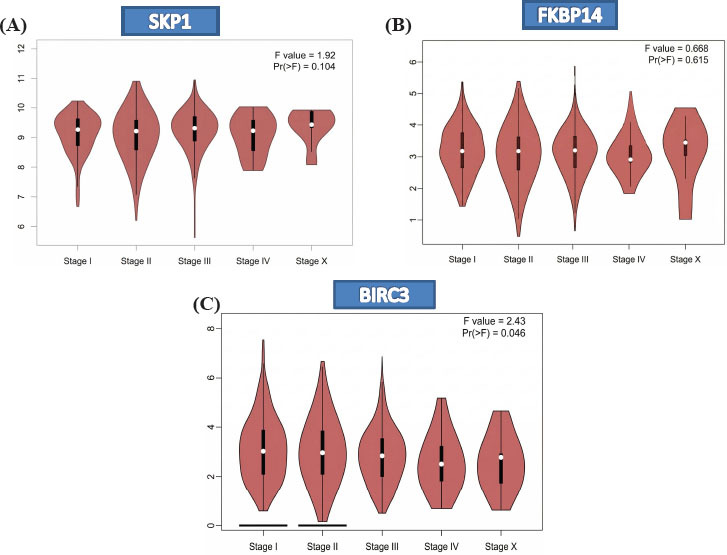
Stage-wise expression of correlation in Breast Invasive Carcinoma (BRCA) patients from GEPIA2 Database. SKP1 (**A**), FKBP14 (**B**), BIRC3 (**C**). The figure represents the correlation between SKP1, FKBP14, and BIRC3 expression levels in different stages (stage I, II, III, IV, X of Breast invasive carcinoma (BRCA). The box illustrates the interquartile range, median expression levels, and outliers.

The expression levels of various proteins can vary according to cancer stage [43]. To explore the stage-specific expression of the marker genes, the correlation of SKP1, BIRC3, and FKBP14 with clinicopathological features of breast cancer patients was analyzed using the GEPIA2 database. The notation “Pr” in the upper-right corner of the plots represents the probability, and values with (Pr>F) < 0.05 were considered statistically significant. Among the three prognostic proteins, only BIRC3 exhibited a significant difference in expression across different stages of breast cancer (Fig. **2a**-**c**).

### 3.4 Role of SKP1, FKBP14 and BIRC3 in Breast Cancer Prognosis

**Fig. (3):**
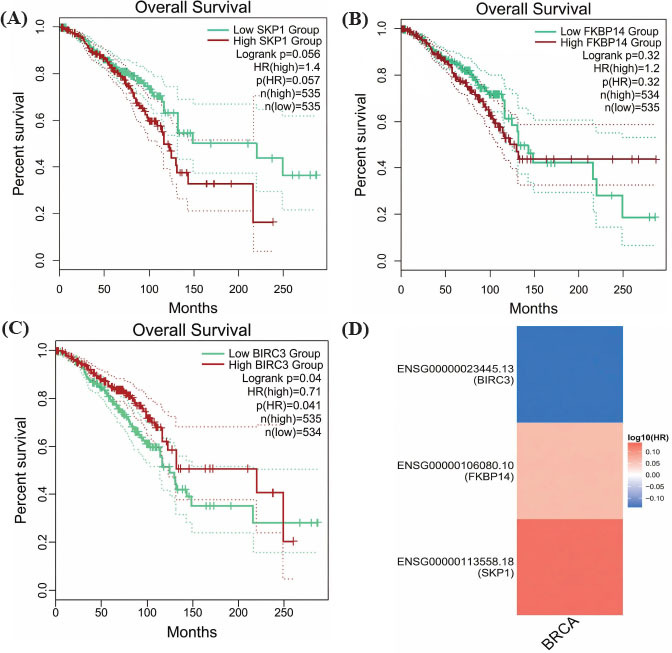
Kaplan-Meier curves for overall survival in BRCA patients with low and high SKP1 (**A**), FKBP14 (**B**), and BIRC3 (**C**) expression obtained from GEPIA2; GEPIA2, gene expression profiling interactive analysis 2; HR, hazard ratio; KM, Kaplan-Meier (**D**) The heat map depicts the hazard ratios in logarithmic scale for different genes. The red block denotes higher risk, while the blue shows less risk.

A Kaplan-Meier curve was generated to assess survival based on the expression levels of the three prognostic genes. High- and low-expression cohorts were defined using 50% cutoff thresholds. Survival analysis was performed using hazard ratios with a 95% confidence interval as the outcome measure. The Y-axis represents the percentage of patients alive, and the X-axis indicates follow-up time in months. The green curve represents low-expression patients, while the red curve represents high-expression patients. Sample sizes were equal for both expression groups. Survival comparisons across TCGA tumor types revealed that lower SKP1 expression (*p* = 0.05, Fig. **3a**) and higher BIRC3 expression (*p* = 0.04, Fig. **3c**) were associated with better overall survival and prognosis in breast cancer. No significant relationship was observed between FKBP14 expression and overall survival (Fig. **3b**).

A heat map depicting hazard ratios on a logarithmic scale further highlighted risk levels, with red indicating higher risk and blue indicating lower risk. SKP1 was identified as highly hazardous, correlating with poorer patient survival outcomes (Fig. **3d**).

### 3.5 Correlation Analysis

**Fig. (4):**
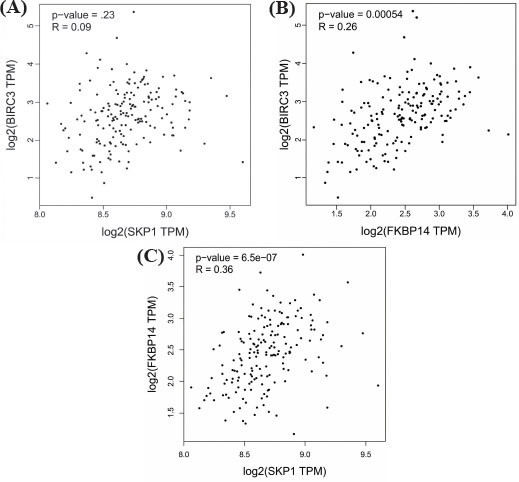
Correlation analysis of gene expression correlation between selected gene pairs using the GEPIA2 website. Figures represent the correlation between (**a**) BIRC3 – SKP1, (**b**) BIRC3 – FKBP14 (**c**) FKBP14 – SKP1 expression in Breast invasive Carcinoma (BRCA) patients.

We further tried to look at the existing correlation between the expression of these genes. Pairwise gene expression correlation analysis used the selected genes' Pearson correlation coefficient and TCGA and GTEx data. Pearson coefficient was calculated automatically and revealed a significant correlation between BIRC3 and FKBP14 (*P*=0.00054) and FKBP14 and SKP1 (*P* = 6.5e-07) and a non-significant correlation between BIRC3 and SKP1 (*p*=0.23). The correlation coefficient graph of BIRC3 and SKP1, FKBP14 and BIRC3, SKP1 and FKBP14 is shown in Fig. **4a**-**c**.

### 3.6 Association between Expression of ER Stress-associated Prognostic Markers and Clinicopathological Parameters in Breast Cancer

Several etiological factors, including genetics, environment, lifestyle, age, and race, influence breast cancer prognosis and, consequently, the effectiveness of treatment options [44, 45]. We assessed the associations of SKP1, FKBP14, and BIRC3 expression with sex, age, ethnicity, breast cancer subclass, nodal metastasis, and menopause status using UALCAN. Statistical significance was determined using the log-rank test (*p*-value).

For SKP1, significant differences in expression were observed across racial groups: (a) normal *vs*. Caucasian, (b) normal *vs*. African American, (c) Caucasian *vs*. African American, and (d) African American *vs*. Asian. Regarding nodal metastasis, significant pairwise differences were noted between (a) normal *vs*. N1, (b) normal *vs*. N2, (c) N0 *vs*. N1, and (d) N0 *vs*. N2. Age-wise, SKP1 expression differed significantly between normal tissue and patients aged 61–80 years and 81–100 years. Gender-based analysis revealed a significant difference between normal and female patients, while menopause status showed significant associations in (a) normal *vs*. post-menopause and (b) pre-menopause *vs*. post-menopause.

For BIRC3, no significant differences were observed across race and age groups. However, gender-based differences were significant: (a) normal *vs*. male and (b) male *vs*. female. Menopause status also showed significance in pre-menopause *vs*. post-menopause comparisons.

**Fig. (5):**
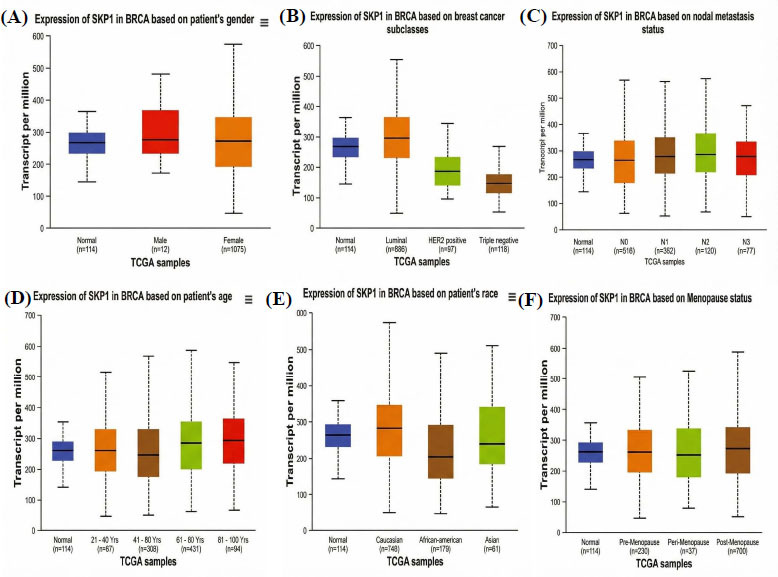
The SKP1 mRNA expression in BRCA patients analyzed in the UALCAN database based on the patient’s gender (**A**), subclass (**B**), nodal metastasis status (**C**), age (**D**), race (**E**), menopause status (**F**).

**Fig. (6):**
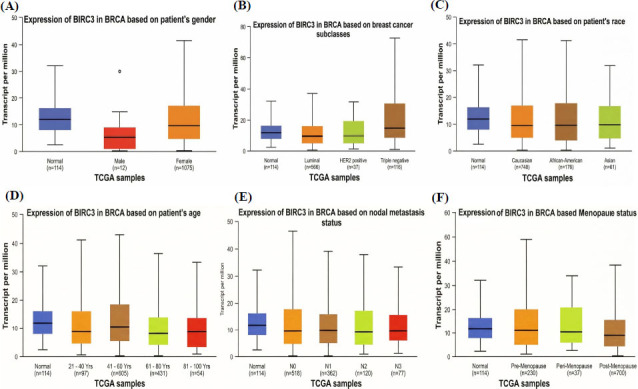
The BIRC3 mRNA expression in BRCA patients analyzed in the UALCAN database based on the patient’s gender (**A**), subclass (**B**), nodal metastasis status (**C**), age (**D**), race (**E**), menopause status (**F**).

**Fig. (7):**
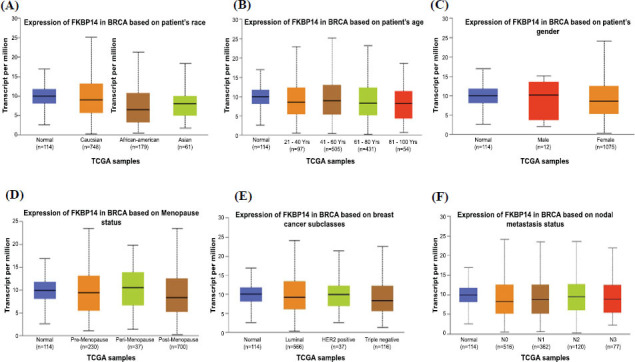
The FKBP14 mRNA expression in BRCA patients analyzed in the UALCAN database based on the patient’s gender (**A**), subclass (**B**), nodal metastasis status (**C**), age (**D**), race (**E**), menopause status (**F**).

FKBP14 expression did not show significant differences across gender, age, or menopause status. However, race-based differences were significant: (a) normal *vs*. African American, (b) Caucasian *vs*. African American, and (c) African American *vs*. Asian. Regarding nodal metastasis, significant differences were observed in N0 *vs*. N1 and N0 *vs*. N3 comparisons (Fig. **5**-**7a**-**f**).

### 3.7 Analysis of the Level of Promoter Methylation and Genomic Alterations

**Fig. (8):**
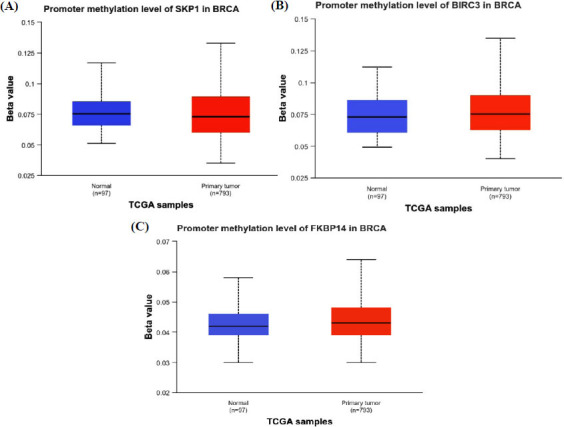
Validation of DNA methylation level using the online tool UALCAN. SKP1 (**A**), FKBP12 (**B**), and BIRC3 (**C**) methylation levels were also significantly decreased in breast cancer patients compared to normal controls.

The promoter methylation levels of SKP1, FKBP14, and BIRC3 were assessed using the UALCAN tool with data from the TCGA dataset. DNA methylation was quantified using beta values ranging from 0 to 1, where values of 0.5–0.7 indicate hypermethylation and 0.25–0.3 indicate hypomethylation. Analysis revealed that the methylation levels of these three prognostic markers were significantly lower in normal tissues compared to breast cancer tissues (Fig. **8a**-**c**), suggesting that promoter methylation of SKP1, FKBP14, and BIRC3 may contribute to breast cancer progression.

### 3.8 Expression of BIRC3 in Different Stages of Breast Cancer

BIRC3 has been shown to play important roles in both pro-survival and tumor-suppressive functions across different cancer types. Genetic inactivation of BIRC3, due to deletions or point mutations, is associated with shorter progression-free survival and poorer prognosis in chronic lymphocytic leukemia (CLL). Conversely, gliomas exhibit increased BIRC3 expression, which correlates with progression from low- to high-grade tumors, highlighting its pro-survival role [46].

A novel function of BIRC3 in stemness reprogramming of glioblastoma (GBM) has also been reported, suggesting that BIRC3 promotes stemness and self-renewal in GBM. Specifically, BIRC3 enhances stemness reprogramming in human and mouse cell lines, as well as patient-derived GBM stem cells (GSCs), through regulation of BMP4 signaling pathways. These findings indicate that targeting BIRC3 could serve as a therapeutic strategy to disrupt stemness and self-renewal, potentially improving treatment outcomes in GBM patients [47].

**Fig. (9):**
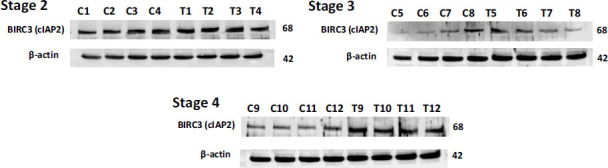
Western blot analysis of various control adjacent tissues *vs* cancerous tissue was performed with BIRC3 antibody. Here, the C1 to C4 represents the control adjacent cells to the cancerous cells at stage 2, while T1 to T4 represents tumor cells at stage 2. Similarly, C5-C8 represents the control adjacent cells to the cancerous cells at stage 3 while T5 to T8 represents tumor cells at stage 3 and C9-C12 represents the control adjacent cells to the cancerous cells at stage 4 while T9 to T12 represents tumor cells at stage 4. Beta-actin was used as an internal control.

In breast cancer, BIRC3 expression was analyzed in tumor samples from stages II, III, and IV, alongside adjacent control tissues. Western blot analysis revealed that BIRC3 expression was higher in tumor tissues compared to corresponding adjacent controls across all stages (Fig. **9**). This elevated expression further supports the bioinformatic analysis, reinforcing BIRC3 as a prognostic marker and a potential therapeutic target in breast cancer.

## 4 DISCUSSION

The accumulation of unfolded or misfolded proteins in the lumen of the endoplasmic reticulum (ER) leads to a condition known as ER stress, which triggers a cytoprotective signaling cascade called the unfolded protein response (UPR) [48]. Downstream mediators of the UPR work to restore and maintain ER homeostasis, but if the stress remains unresolved, apoptosis is induced [49]. Three transmembrane receptors, PKR-like kinase (PERK), inositol-requiring enzyme 1 (IRE1), and activating transcription factor 6 (ATF6), sense disturbances in ER function [50]. Activation of these receptors initiates the three branches of UPR signaling, which collectively attempt to restore ER homeostasis but drive cells toward apoptosis if the stress persists. Tumor initiation, growth, and limited vascularization increase cellular stress, prompting cells to activate multiple stress response pathways, including UPR, to enhance ER folding capacity and prevent apoptosis [51].

Several studies have highlighted the intricate relationship between ER stress and cancer. The rising incidence and mortality of breast cancer underscore the need for novel predictive biomarkers to improve diagnosis and prognosis. In this study, three ER stress-associated genes, SKP1, FKBP14, and BIRC3, were identified as potential prognostic biomarkers for breast cancer. UPR-associated genes were initially extracted from STRING and Harmonizome Reactome pathway databases. While these sources provide well-annotated pathways, the completeness of the gene list cannot be fully assured as databases are continually updated with new discoveries. Future studies incorporating experimental validation, extended time frames, and additional datasets may further refine this gene list and provide additional insights.

By comparing RNA sequencing expression data, we observed that SKP1 and FKBP14 are upregulated in tumor tissues relative to normal adjacent tissues, whereas BIRC3 expression did not show a significant change. Stage-specific analysis using the GEPIA2 database revealed that BIRC3 expression decreases as the tumor progresses through various stages. However, Western blot analysis of tumor *versus* adjacent control tissues across different stages demonstrated that BIRC3 expression is higher in tumor tissues at all stages, strongly supporting its potential as a predictive biomarker in breast cancer progression. Survival analysis indicated that higher SKP1 expression correlates with better overall survival, while higher BIRC3 expression is linked to poorer survival outcomes. These findings are consistent with the initial bioinformatic results (Table **1**), confirming SKP1 as a marker of favorable prognosis and BIRC3 as a marker of poor prognosis.

Our data also indicate a significant correlation between FKBP14 and SKP1, and all three proteins show differential expression across race, gender, age, and disease stage. Additionally, we observed evidence of epigenetic regulation, as the promoters of all three genes were hypermethylated in tumor cells compared to adjacent normal tissues. Overall, our study strongly supports the use of BIRC3 as a prognostic marker for different stages of breast cancer, providing insight into future prognosis and overall survival. Similarly, SKP1 may serve as a predictor of overall survival and disease progression.

## CONCLUSION

BIRC3, FKBP14, and SKP1 have been shown to play important roles in clinical contexts. BIRC3 is implicated in several malignancies, including gliomas, chronic lymphocytic leukemia, and esophageal cancer, and its expression levels can influence tumor behavior and patient prognosis. In colorectal cancer, increased BIRC3 expression is associated with resistance to chemotherapeutic agents. FKBP14, a member of the FKBP family, is involved in protein folding and trafficking, though its precise role in cancer remains less well defined. SKP1, as a component of the E3 ubiquitin ligase complex, is crucial for regulating protein degradation, and targeting SKP1 may offer a potential therapeutic strategy in cancer. Comprehensive research is required to fully elucidate the clinical significance of FKBP14, BIRC3, and SKP1.

It is important to note that our conclusions are based on data derived from public databases, which introduces certain limitations. Updates or modifications in these databases over time may affect the observed relationships between these prognostic markers and cancer. Therefore, further experimental validation is necessary to confirm these findings.

## Figures and Tables

**Table 1:** Endoplasmic reticulum stress associated genes as prognostic markers in breast cancer.

**Genes**	**Log-rank *P* value**	**Favorable/unfavourable**
SKP1	0.00075	Unfavorable
BIRC3	0.00036	Favorable
FKBP14	0.00016	Unfavorable

## Data Availability

The authors confirm that the data supporting the findings of this research are available within the article.

## LIST OF ABBREVIATIONS

ATF6: Activating Transcription Factor 6
BIRC3: Baculoviral IAP Repeat Containing 3
ER: Endoplasmic Reticulum
FKBP14: FK506-Binding Protein 14
IRE1: Inositol Requiring Enzyme 1
PERK: Protein Kinase R-Like Kinase
SKP1: S-Phase Kinase-Associated Protein 1
UPR: Unfolded Protein Response

## STANDARDS OF REPORTING

Strobe guidelines were followed.
