# Supplementary material for: Analysis of Endoplasmic Reticulum Stress-Associated Proteins As Prognostic Markers In Breast Cancer
Source: Curr Genomics. 2026 Jan 6;26(6):621–32. doi: 10.2174/0113892029374158251030064845 (PMC13154239; doi:10.2174/0113892029374158251030064845)
Supplement: Supplementary file 1 [file CG-26-6-621_SD1.pdf]

Supplementary Material

Analysis of Endoplasmic Reticulum Stress-Associated Proteins As Prognostic Markers In Breast Cancer

Smriti Shreya[1], Shweta Pandey[2], Debasish Kumar Ghosh[3], Puneet[4], Shyam Babu Prasad[5], Christophe F. Grosset[6] and Buddhi Prakash Jain[1,*]

[1]Gene Expression and Signaling Lab, Department of Zoology, Mahatma Gandhi Central University, Motihari, Bihar, India; [2]Department of Biotechnology, Govt Vishwanath Yadav Tamaskar Post-Graduate Autonomous College, Durg, Chhattisgarh, India; [3]Kasturba Medical College, Manipal Academy of Higher Education, Manipal, Karnataka 576104, India; [4]Department of General Surgery, Institute of Medical Sciences, Banaras Hindu University, Varanasi, Uttar Pradesh, India; [5]Department of Zoology, Mahatma Gandhi Central University, Motihari, Bihar, India; [6]MIRCADE Team, U1312, Bordeaux Institute in Oncology, BRIC, Université de Bordeaux, 146 Rue Léo Saignat, F-33000 Bordeaux, France

Supplementary Table 1.

S. No.	Criteria	Range	Numbers of Sample
1	Gender	Normal<br>Male<br>Female	114<br>12<br>1075
2	Subclass	Luminal<br>Her + ve<br>Triple Negative Breast Cancer	566<br>37<br>116
3	Nodal metastasis	N0<br>N1<br>N2<br>N3	561<br>362<br>120<br>77
4	Age	21-40 years<br>41-60 years<br>61-80 years<br>81-100 years	97<br>505<br>431<br>54
5	Race	Caucasian<br>African-American<br>Asian	748<br>179<br>61
6	Menopause	Pre-menopause<br>Peri-menopause<br>Post-menopause	230<br>37<br>700

Supplementary Table 2. List of ER stress associated genes and their functions (By Harmonizome and Human Protein Atlas).

Gene description	Gene name	Functions
Heat shock protein family A (Hsp70) member 5	BiP/GRP78	Involved in the correct folding of proteins and degradation of misfolded proteins
Calreticulin	CALR	Functions in protein folding quality control and calcium homeostasis
Calnexin	CANX	calcium-binding, endoplasmic reticulum (ER)-associated protein that interacts transiently with newly synthesized N-linked glycoproteins, facilitating protein folding and assembly
Protein disulfide isomerase family A member 3	PDIA3	Catalyzes the formation, isomerization, and reduction or oxidation of disulfide bonds
Protein disulfide isomerase family A member 4	PDIA4	Catalyze protein folding and thiol-disulfide interchange reactions
Protein disulfide isomerase family A member 5	PDIA5	Catalyze protein folding and thiol-disulfide interchange reactions
Glucosidase II alpha subunit	GANAB	Cleaves sequentially the 2 innermost alpha-1,3-linked glucose residues from the Glc(2)Man(9)GlcNAc(2) oligosaccharide precursor of immature glycoproteins
Hypoxia up-regulated 1	HYOU1	pivotal role in cytoprotective cellular mechanisms triggered by oxygen deprivatio
Endoplasmic reticulum to nucleus signaling 1	IRE1 alpha	key sensor for the endoplasmic reticulum unfolded protein response (UPR)
Endoplasmic reticulum to nucleus signaling 2	IRE1 beta	Induces translational repression through 28S ribosomal RNA cleavage in response to ER stress. Pro-apoptotic.
Phosphoinositide-3-kinase regulatory subunit 1	PIK3R1	Necessary for the insulin-stimulated increase in glucose uptake and glycogen synthesis in insulin-sensitive tissue. promoting nuclear translocation of XBP1 isoform 2.
X-box binding protein 1	XBP1	Functions as a transcription factor during endoplasmic reticulum (ER) stress. Lipid biosynthesis, Lipid metabolism,
Mitogen-activated protein kinase kinase kinase 5	ASK1	essential component of the MAP kinase signal transduction pathway
DnaJ heat shock protein family (Hsp40) member B9	DNAJB9	induced by endoplasmic reticulum stress and plays a role in protecting stressed cells from apoptosis
ER degradation enhancing alpha-mannosidase like protein 1	EDEM1	directly involved in endoplasmic reticulum-associated degradation (ERAD) and targets misfolded glycoproteins for degradation in an N-glycan-independent manner.
Mitogen-activated protein kinase 8	JNK1	act as an integration point for multiple biochemical signals
Mitogen-activated protein kinase kinase kinase 5	MAP3K5	role in the apoptosis signal transduction pathway through mitochondria-dependent caspase activation.

TNF receptor associated factor 2	TRAF2	Regulates activation of NF-kappa-B and JNK and plays a central role in the regulation of cell survival and apoptosis
Presenilin 1	PSEN1	Plays a role in Notch and Wnt signaling cascades and regulation of downstream processes
Presenilin 2	PSEN2	modulates calcium ions shuttling between ER and mitochondria
Receptor for activated C kinase 1	RACK1	Scaffolding protein involved in the recruitment, assembly and/or regulation of a variety of signaling molecules.
Protein disulfide isomerase family A member 6	PDIA6	Function as a chaperone that inhibits aggregation of misfolded proteins
Eukaryotic translation initiation factor 2 alpha kinase 3	PERK	Key activator of the integrated stress response (ISR) required for adaptation
Eukaryotic translation initiation factor 2A	EIF2A	Functions in the early steps of protein synthesis of a small number of specific mRNAs
Eukaryotic translation initiation factor 2 alpha kinase 1	EIF2AK1	acts as a key sensor of heme-deficiency
Eukaryotic translation initiation factor 2 alpha kinase 2	EIF2AK2	plays a key role in the innate immune response to viral infection
Eukaryotic translation initiation factor 2 alpha kinase 4	EIF2AK4	phosphorylates the alpha subunit of EIF2S1/eIF-2-alpha in response to low amino acid availability
Eukaryotic translation initiation factor 2 subunit alpha	EIF2S1	Functions in the early steps of protein synthesis by forming a ternary complex with GTP and initiator tRNA
Eukaryotic translation initiation factor 2 subunit beta	EIF2S2	Helps in protein synthesis by forming a ternary complex with GTP and initiator tRNA
Eukaryotic translation initiation factor 2 subunit gamma	EIF2S3	involved in the early steps of protein synthesis.
Activating transcription factor 3	ATF3	Represses transcription from promoters with ATF sites.
Activating transcription factor 4	ATF4	regulator of metabolic and redox processes under normal cellular conditions, and as master transcription factor during integrated stress response (ISR)
Tribbles pseudokinase 3	TRIB3	Inhibits the transcriptional activity of DDIT3/CHOP
DNA damage inducible transcript 3	CHOP	induces cell cycle arrest and apoptosis in response to ER stress
Protein phosphatase 1 regulatory subunit 15A	GADD34	prevents excessive phosphorylation of the translation initiation factor eIF-2A.
Protein phosphatase 1 catalytic subunit beta	PPP1CB	participates in the regulation of glycogen metabolism, muscle contractility and protein synthesis.
Protein phosphatase 1 catalytic subunit gamma	PPP1CC	participates in the regulation of glycogen metabolism, muscle contractility and protein synthesis.
Endoplasmic reticulum oxidoreductase 1 alpha	ERO1 alpha	involved in disulfide bond formation in the endoplasmic reticulum

Endoplasmic reticulum oxidoreductase 1 beta	ERO1 beta	Enables thiol oxidase activity. Involved in protein folding in endoplasmic reticulum.
Activating transcription factor 5	ATF5	stimulates or represses gene transcription through binding of different DNA regulatory elements
Homocysteine inducible ER protein with ubiquitin like domain 1	HERPud1	involved in ubiquitin-dependent degradation of misfolded endoplasmic reticulum proteins
Heat shock protein 90 beta family member 1	GRP94/HSP90B1	Helps in the processing and transport of secreted proteins
Nuclear factor, erythroid 2 like 1	NFE2L1	Constitutes a precursor of the transcription factor NRF1
Nuclear factor, erythroid 2 like 2	NFE2L2	plays a key role in the response to oxidative stress.
Baculoviral IAP repeat containing 2	BIRC2	modulates inflammatory signaling and immunity, mitogenic kinase signaling, and cell proliferation, as well as cell invasion and metastasis
Baculoviral IAP repeat containing 3 ULK1	BIRC3	also modulates inflammatory signaling and immunity, mitogenic kinase signaling and cell proliferation, as well as cell invasion and metastasis.
Unc-51 like autophagy activating kinase 1	ULK1	involved in autophagy in response to starvation
Lysosomal associated membrane protein 3	LAMP3	plays a role in the unfolded protein response (UPR) that contributes to protein degradation and cell survival during proteasomal dysfunction
Activating transcription factor 6	ATF6	Precursor of the transcription factor form
Activating transcription factor 6 beta	ATF6 beta	transcription factor in the unfolded protein response (UPR) pathway during ER stress.
Membrane bound transcription factor peptidase, site 1	MBTPS1	Helps in Cholesterol metabolism, Lipid metabolism, Steroid metabolism.
Membrane bound transcription factor peptidase, site 2	MBTPS2	mediates intramembrane proteolysis of proteins
DnaJ heat shock protein family (Hsp40) member A3	DNAJA3	Modulates apoptotic signal transduction or effector structures within the mitochondrial matrix
DnaJ heat shock protein family (Hsp40) member B1	DNAJB1	Interacts with HSP70 and can stimulate its ATPase activity.
DnaJ heat shock protein family (Hsp40) member B2	DNAJB2	Functions as a co-chaperone
DnaJ heat shock protein family (Hsp40) member B6	DNAJB6	acts as a co-chaperone of HSP70
DnaJ heat shock protein family (Hsp40) member B9	DNAJB9	Acts as co-chaperone for Hsp70 protein HSPA5/BiP that acts as a key repressor of the ERN1/IRE1-mediated UPR
DnaJ heat shock protein family (Hsp40) member B11	DNAJB11	Acts as co-chaperone for HSPA5 it is required for proper folding, trafficking or degradation of proteins
ily (Hsp40) member B12	DNAJB12	promote protein folding and trafficking, prevent aggregation of client proteins, and promote unfolded proteins to endoplasmic reticulum-associated degradation (ERAD) pathway

DnaJ heat shock protein family (Hsp40) member B14	DNAJB14	Acts as a co-chaperone with HSPA8/Hsc70
DnaJ heat shock protein family (Hsp40) member C1	DNAJC1	modulate protein synthesi
DnaJ heat shock protein family (Hsp40) member C3	DNAJC3	acts as a negative regulator of the EIF2AK4/GCN2 kinase activity by preventing the phosphorylation of eIF-2-alpha
DnaJ heat shock protein family (Hsp40) member C5	DNAJC5	Acts as a general chaperone in regulated exocytosis
DnaJ heat shock protein family (Hsp40) member C5 beta	DNAJC5B	Acts as Chaperone
DnaJ heat shock protein family (Hsp40) member C7	DNAJC7	Acts as co-chaperone regulating the molecular chaperones
DnaJ heat shock protein family (Hsp40) member C10	DNAJC10	Helps in correct folding of proteins and degradation of misfolded proteins
DnaJ heat shock protein family (Hsp40) member C12	DNAJC12	act as a co-chaperone for HSP70
DnaJ heat shock protein family (Hsp40) member C21	DNAJC21	act as a co-chaperone for HSP70, rRNA biogenesis
Exosome component 1	EXOSC1	Participates in rRNA processing
Exosome component 2	EXOSC2	Participates in rRNA processing
Exosome component 3	EXOSC3	Participates in rRNA processing
Exosome component 4	EXOSC4	Participates in rRNA processing
Exosome component 5	EXOSC5	Participates in rRNA processing
Exosome component 6	EXOSC6	Participates in rRNA processing
Exosome component 7	EXOSC7	Participates in rRNA processing
Exosome component 8	EXOSC8	Participates in rRNA processing
Exosome component 9	EXOSC9	Participates in rRNA processing
Mitogen-activated protein kinase 14	MAPK14	essential component of the MAP kinase signal transduction pathway
Calumenin	CALU	inhibit gamma-carboxylase GGCX. Binds 7 calcium ions with a low affinity
Peptidylprolyl isomerase B	PPIB	assist protein folding
Prolyl 4-hydroxylase subunit beta	P4HB	catalyzes the formation, breakage and rearrangement of disulfide bonds
Asparagine synthetase (glutamine-hydrolyzing)	ASNS	Helps in Amino-acid biosynthesis, Asparagine biosynthesis
Protein phosphatase 2 regulatory subunit B'beta	PPP2R5B	Acts as regulatory component of the serine/threonine-protein phosphatase 2A
C-C motif chemokine ligand 2	CCL2	Acts as a ligand for C-C chemokine receptor CCR2
WD repeat domain, phosphoinositide interacting 1	WIPI1	controls the major intracellular degradation process
KDEL endoplasmic reticulum protein retention receptor 3	KDELR3	Helps in ER-Golgi transport, Protein transport,
SHC adaptor protein 1	SHC1	Helps in Angiogenesis, Growth regulation

Tripeptidyl peptidase 1	TPP1	act as a non-specific lysosomal peptidase which generates tripeptides from the breakdown products produced by lysosomal proteinases
Heparin binding growth factor	HDGF	Acts as a transcriptional repressor.
Talin 1	TLN1	involved in connections of major cytoskeletal structures to the plasma membrane
Exostosin like glycosyltransferase 3	EXTL3	Helps in biosynthesis of heparan sulfate (HS)
TSPY like 2	TSPYL2	modulates gene expression in response to neuronal synaptic activity,
Dynactin subunit 1	DCTN1	Plays a key role in dynein-mediated retrograde transport of vesicles and organelles
Sulfotransferase family 1A member 4	SULT1A4	Helps in Catecholamine metabolism, Lipid metabolism, Steroid metabolism
Poly(A)-specific ribonuclease	PARN	Helps in Nonsense-mediated mRNA decay
Adducin 1	ADD1	Helps in Actin-binding, Calmodulin-binding
Zinc finger and BTB domain containing 17	ZBTB17	function as an activator or repressor depending on its binding partners
Acyl-CoA dehydrogenase very long chain	ACADVL	catalyze the first step of mitochondrial fatty acid beta-oxidation
SEC31 homolog A, COPII coat complex component	SEC31A	promotes the formation of transport vesicles from the endoplasmic reticulum (ER)
Signal sequence receptor subunit 1	SSR1	bind calcium to the ER membrane
Signal sequence receptor subunit 2	SSR2	bind calcium to the ER membrane and thereby regulate the retention of ER resident proteins
ADP ribosylation factor GTPase activating protein 1	ARFGAP1	Involved in membrane trafficking and /or vesicle transport
ATPase H+ transporting V0 subunit d1	ATP6V0D1	Helps in Cilium biogenesis/degradation, Hydrogen ion transport,
Prolactin regulatory element binding	PREB	activates the small GTPase SAR1B
Lamin A/C	LMNA	provide a framework for the nuclear envelope and may also interact with chromatin
FK506 binding protein 14	FKBP14	accelerates the folding of proteins during protein synthesis
Stress associated endoplasmic reticulum protein 1	SERP1	Interacts with target proteins during their translocation into the lumen of the ER.
SRP receptor beta subunit	SRPRB	Component of the signal recognition particle (SRP) complex receptor.
CTD small phosphatase 2	CTDSP2	catalyzes the dephosphorylation of 'Ser-5' within the tandem 7 residue repeats in the C-terminal domain (CTD) of the largest RNA polymerase II subunit POLR2A
Golgi SNAP receptor complex member 2	GOSR2	Involved in transport of proteins from the cis/medial-Golgi to the trans-Golgi network
Yip1 interacting factor homolog A, membrane trafficking protein	YIF1A	Helps in ER-Golgi transport, Protein transport

DEAD/H-box helicase 11	DDX11	Helps in DNA replication, DNA repair and heterochromatin organization as well as in ribosomal RNA synthesis
Wolframin ER transmembrane glycoprotein	WFS1	Maintain cellular Ca(2+) homeostasis
Synoviolin 1	SYVN1	Role in Stress response, Ubl conjugation pathway
Kelch domain containing 3	KLHDC3	Role in Ubl conjugation pathway
DIS3 homolog, exosome endoribonuclease and 3'-5' exoribonuclease	DIS3	Helps in rRNA processing
CXXC finger protein 1	CXXC1	Heps in Transcription regulation
KH-type splicing regulatory protein	KHSRP	role in mRNA trafficking
Cullin 7	CUL7	Ubl conjugation pathway
Insulin like growth factor binding protein 1	IGFBP1	inhibit or stimulate the growth promoting effects of the IGFs
Glycogen synthase kinase 3 alpha	GSK3A	Helps in Carbohydrate metabolism, Glycogen metabolism, Neurogenesis,
Nuclear transcription factor Y subunit beta	NFYB	Helps in Transcription regulation
TatD DNase domain containing 2	TATDN2	Putative deoxyribonuclease.
Decapping mRNA 2	DCP2	Helps in Decapping metalloenzyme
Polycystin 2, transient receptor potential cation channel	PKD2	Helps in Calcium transport, Ion transport, Potassium transport
Lectin, mannose binding 1	LMAN1	ER-Golgi transport, Protein transport
Endoplasmic reticulum protein 29	ERP29	important role in the processing of secretory proteins
Thioredoxin domain containing 5	TXNDC5	involved in the formation of disulfide bonds in proteins
Exostosin like glycosyltransferase 2	EXTL2	required for the biosynthesis of heparan-sulfate and responsible for the alternating addition of beta GlcA and GlcNAc
Endoplasmic reticulum to nucleus signaling 1	ERN1	key sensor for the unfolded protein response (UPR)
Vasodilator stimulated phosphoprotein	VASP	involved in a range of processes dependent on cytoskeleton remodeling and cell polarity
Sorbin and SH3 domain containing 3	SORBS3	plays a role in cell spreading and enhances the activation of JNK/SAPK.
Vinculin	VCL	involved in cell-matrix adhesion and cell-cell adhesion
SOS Ras/Rho guanine nucleotide exchange factor 2	SOS2	Promotes the exchange of Ras-bound GDP by GTP
Golgi SNAP receptor complex member 1	GOSR1	Helps in ER-Golgi transport, Protein transport
Glutamine--fructose-6-phosphate transaminase 1	GFPT1	Controls the flux of glucose into the hexosamine pathway.

ATPase H+ transporting V1 subunit B1	ATP6V1B1	Helps in Hydrogen ion transport, Ion transport
ATPase H+ transporting V1 subunit D	ATP6V1D	Cilium biogenesis/degradation, Hydrogen ion transport,
ATPase H+ transporting V0 subunit d1	ATP6V0D1	Helps in Cilium biogenesis/degradation, Hydrogen ion transpor
Exostosin like glycosyltransferase 2	EXTL1	required for the biosynthesis of heparan-sulfate (HS)
Mtr4 exosome RNA helicase	MTREX	Catalyzes the ATP-dependent unwinding of RNA duplexes.
Myeloid derived growth factor	MYDGF	Helps in Angiogenesis, Apoptosis
WD repeat domain, phosphoinositide interacting 1	WIPI1	Plays an important role in starvation- and calcium-mediated autophagy, as well as in mitophagy
Phospholipase A2 group IVB	PLA2G4B	Helps in membrane phospholipid remodeling.
Bet1 golgi vesicular membrane trafficking protein	BET1	Helps in vesicular transport from the ER to the Golgi complex
YKT6 v-SNARE homolog	YKT6	Helps in ER-Golgi transport, Protein transport
Dendrocyte expressed seven transmembrane protein	DCSTAMP	roles in cellular fusion, cell differentiation, bone and immune homeostasis.
CREB3 regulatory factor	CREBRF	Represses the transcriptional activity of CREB3 during the UPR
Cereblon	CRBN	Acts as Substrate recognition component of a DCX (DDB1-CUL4-X-box) E3 protein ligase comple
Ring-box 1	RBX1	Helps in DNA damage, DNA repair, Ubl conjugation pathway
S-phase kinase associated protein 1	SKP1	mediates the ubiquitination of proteins involved in cell cycle progression, signal transduction and transcription
Cell division cycle 34, ubiqiutin conjugating enzyme	CDC34	Accepts ubiquitin from the E1 complex and catalyzes its covalent attachment to other proteins.
NOP53 ribosome biogenesis factor	NOP53	Helps in Ribosome biogenesis.
C-X-C motif chemokine ligand 8	CXCL8	plays an important role in neutrophil activation and chemotaxsis.
CAMP responsive element binding protein 3 like 2	CREB3L2	Helps in Transcription regulation.
CAMP responsive element binding protein 3	CREB3	involved in cell proliferation, migration and differentiation, tumor suppression and inflammatory gene expression
CAMP responsive element binding protein 3 like 4	CREB3L4	Transcriptional activator that may play a role in the unfolded protein response
Tripeptidyl peptidase 1	TPPP1	Helps in GTPase activity; magnesium ion binding activity; and protein homodimerization activity
CCAAT enhancer binding protein gamma	CEBPG	Helps in Transcription regulation
CAMP responsive element binding protein 3 like 1	CREB3L1	involved in unfolded protein response (UPR)

Protein phosphatase 1 regulatory subunit 15A	PPP1R15A	prevents excessive phosphorylation of eIF-2A
Eukaryotic translation initiation factor 2 alpha kinase 3	EIF2AK3	Acts as Metabolic-stress sensing protein kinase that phosphorylates the alpha subunit of EIF2S1/eIF-2-alpha.
Solute carrier family 7 member 5	SLC7A5	Helps in Amino-acid transport
ChaC glutathione specific gamma-glutamylcyclotransferase 1	CHAC1	Catalyzes the cleavage of glutathione into 5-oxo-L-proline and a Cys-Gly dipeptide

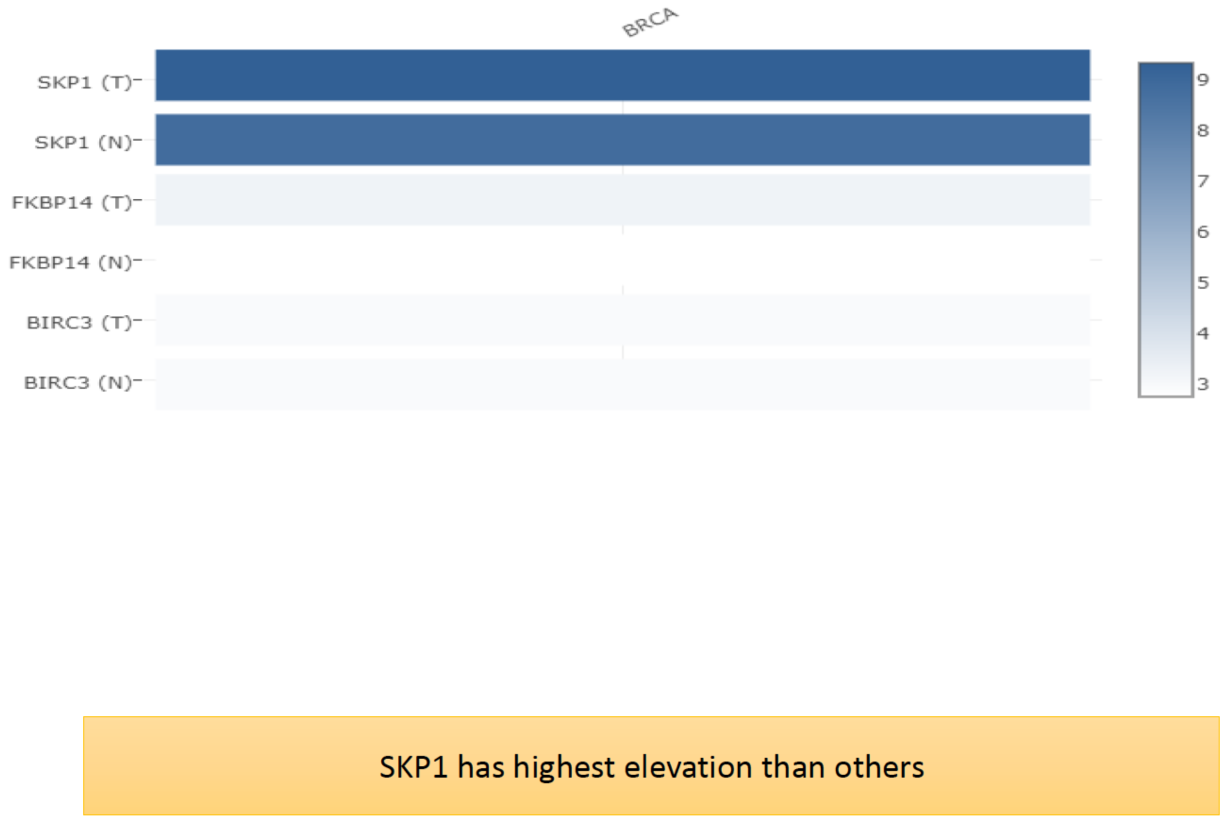

Supplementary Fig. (1). SKP1, FKBP14, and BIRC3 expression level across BRCA TCGA cancer types (GEPIA2) where SKP1 has highest elevation than BIRC3 and FKBP14 in tumor compare to normal tissues.
